# Sulforaphane, an Nrf-2 Agonist, Modulates Oxidative Stress and Inflammation in a Rat Model of Cuprizone-Induced Cardiotoxicity and Hepatotoxicity

**DOI:** 10.1007/s12012-022-09776-0

**Published:** 2023-01-17

**Authors:** Ghadha Ibrahim Fouad

**Affiliations:** Department of Therapeutic Chemistry, Pharmaceutical and Drug Industries Research Institute, National Research Centre, 33 El-Bohouth St., Dokki, Cairo, 12622 Egypt

**Keywords:** Sulforaphane, Cuprizone, Nuclear factor E2 related factor (Nrf-2), Cardiotoxicity, Hepatotoxicity, Interferon-gamma

## Abstract

Cuprizone (CPZ) is a neurotoxic agent that is used to induce demyelination and neurotoxicity in rats. This study aimed to investigate the protective potential of sulforaphane (SF), nuclear factor E2 related factor (Nrf-2) activator, against CPZ-induced cardiotoxicity and hepatotoxicity. Male adult Wistar rats (*n* = 18) were fed with a regular diet or a CPZ-contained diet (0.2%) for four weeks. The rats were divided into three groups (*n* = 6): negative control rats, CPZ-exposed rats, and CPZ + SF treated rats. SF was intraperitoneally administrated (2 mg/kg/day) for two weeks. The anti-inflammatory and anti-oxidative functions of SF were investigated biochemically, histologically, and immunohistochemically. CPZ increased serum levels of cardiac troponin 1 (CTn1), aspartate amino transaminase (AST), alanine amino transaminase (ALT), and alkaline phosphatase (ALP). In addition, serum levels of inflammatory interferon-gamma (IFN-γ), and pro-inflammatory interleukin 1β (IL-1β) were significantly elevated. Moreover, CPZ administration provoked oxidative stress as manifested by declined serum levels of total antioxidant capacity (TAC), as well as, stimulated lipid peroxidation and decreased catalase activities in both cardiac and hepatic tissues. SF treatment reversed all these biochemical alterations through exerting anti-oxidative and anti-inflammatory activities, and this was supported by histopathological investigations in both cardiac and hepatic tissues. This SF-triggered modulation of oxidative stress and inflammation is strongly associated with Nrf-2 activation, as evidenced by activated immunoexpression in both cardiac and hepatic tissues. This highlights the cardioprotective and hepatoprotective activities of SF via Nrf-2 activation and enhancing catalase function.

## Introduction

Cuprizone (oxalic acid bis (cyclohexylidene hydrazide) is a copper-chelating agent that is used experimentally to provoke demyelination pathology similar to that of multiple sclerosis (MS) [1]. Dietary administration of CPZ stimulates copper deficiency [2] and triggers dysfunction of the copper-dependent electron carrier “cytochrome oxidase” which is an essential component of oxidative phosphorylation [3]. Besides enhancing demyelination and glial activation, CPZ was found to cause alterations in the hepatic tissue, as demonstrated by several studies [4–6]. The hepatotoxic and neurotoxic potentials of CPZ could be attributed to a disturbance of cellular respiration, a key mitochondrial function [7]. It was found that a disruption in the enzymatic activities takes place prior to myelin loss during the first days or weeks of CPZ administration [5]. Concerning MS-associated cardiotoxicity, MS patients demonstrated an elevated cardiovascular risk that might be attributed to the impairment of the autonomic control of cardiovascular functions, but the underlying molecular mechanisms are not completely elucidated [8, 9]. Therefore, this study aimed to explore the influence of CPZ-induced MS on cardiovascular health, more interestingly; no study has been published so far concerning CPZ-induced cardiotoxicity.

Copper “Cu” is a vital “catalytic and structural” cofactor in several biochemical mechanisms [10]; several enzymes use Cu as a cofactor for example, superoxide dismutase-1 [11], monoamine oxidase [12], and cytochrome c oxidase assembly protein [13]. CPZ is a Cu-chelator that is toxic to mitochondria [14–16]. During the early stages of exposure to CPZ, a reduction in monoamine oxidase and cytochrome c oxidase in the brain and liver of mice is observed [17] in association with the development of mega-mitochondria in the liver, which is linked to metabolic disruption [15]. Therefore, CPZ is capable of suppressing the enzymatic activity of monoamine oxidase and copper-dependent enzymes such as cytochrome c oxidase [17–19].

Sulforaphane (SF) is a powerful phytochemical found in seeds and sprouts of cruciferous plants such as broccoli, SF is a pleiotropic agent that exhibited promising multifactorial functions against several chronic diseases, including neurodegenerative disorders and cancer [20–24], moreover, numerous studies showed the hepatoprotective and cardioprotective activities of SF [25–29]. SF is an activator of nuclear factor E2 related factor (Nrf-2) [30], which is a transcription factor involved in the adaptive response to several endogenous and exogenous stressors such as oxidative stress; SF targets the activation of the Nrf-2 pathway which regulates the expression of cytoprotective enzymes that exhibited anti-oxidative and detoxification activities [31].

Due to its electrophilic nature, SF enhances nuclear translocalization and accumulation of Nrf-2 and mediates its phosphorylation by activating different kinases including mitogen-activated protein kinase (MAPK), protein kinase C (PKC), and Akt kinase (protein kinase B or PKB), where it changes nuclear and cytoplasmic trafficking and Nrf-2 integrity and stability [32–34]. Furthermore, SF is considered “an indirect anti-oxidant” that is capable of exerting anti-oxidative potential through modification of Keap1 cysteine residues, activation of MAPK, phosphatidylinositol 3-kinase (PI3K), and PKC pathways, which results in the phosphorylation, nuclear accumulation, and increased transcription and stability of Nrf-2 [35].

The protective potential of SF was evidenced in several studies; however, the protective potential of SF on CPZ-induced cardiotoxicity and hepatotoxicity lacked clarification. The main target of this study is to evaluate the cardioprotective and hepatoprotective potentials of Sulforaphane (SF) in a rat model of CPZ-induced cardiotoxicity and hepatotoxicity.

## Materials and Methods

### Materials

DL-Sulforaphane (SF) (CAS No. 4478-93-7), and Cuprizone (CPZ) (CAS No. 370-81-0) were bought from Sigma, USA. SF was dissolved in 250 μl of Tween 80 (Sigma, St. Louis, MO, USA) and diluted to the appropriate concentration with distilled water; the final concentration of Tween 80 did not exceed 1%. Serum levels of cardiac troponin-1 (CTn1), interferon-γ (IFN-γ), and interleukin 1β (IL-1β) were estimated using commercially available enzyme-linked immunosorbent assay (ELISA) kits bought from (BioVision, USA, Catalog number: E4737; Quantikine, USA, Catalog number RIF00; and BioVision, USA, Catalog number K4796-100, respectively). Colorimetric Kits of malondialdehyde (MDA), catalase, and total antioxidants (TAC) were bought from Biodiagnostic *Co.*, Egypt. Kits of primary Anti-Nrf-2 antibodies (GTX103322) were bought from Gene Tex *Co.*, USA and secondary antibody HRP Envision kit (DAKO) was bought from Agilent, USA. All other chemicals used in the study were of high analytical grade.

### Animals and Grouping

The experiments were performed on male adult Wistar rats (*n* = 18) weighing around 120 ± 20 g and aged 6 weeks from the Animal House of the National Research Center (NRC), Egypt. The animals were kept under suitable laboratory conditions throughout the period of investigation. Animals were kept with free access to food and fresh water in a room with temperatures ranging from 22 to 24 °C and a 12-h light/dark cycle. They were fed standard pellet chow, provided by the animal house at the National Research Centre (NRC), and allowed free access to water. The experiment was conducted in accordance with the National Research Council’s Guide for the Care and Use of Laboratory Animals (NIH Publications No. 8023, revised 1978), and experimental procedures were approved by the Medical Research Ethics Committee (MREC) of the NRC, Egypt (19-313).

### Rat Model of CPZ-Induced Cardiotoxicity and Hepatotoxicity

CPZ-induced toxicity was induced by administrating rats a CPZ-diet prepared by carefully mixing 0.2% of CPZ into ground rodent chow for four weeks, according to Omotoso et al. [36].

### Experimental Design and Treatments

The experimental period lasted for six weeks, including four weeks (30 days) for CPZ intoxication and two weeks (14 days) for treatment with SF. After an acclimatization period of two weeks, the 18 rats were randomly divided into three groups (6 rats/group) as follows:

**Group (1)** Healthy negative control group fed on a normal ground rodent chow.

**Group (2)** CPZ-exposed control group: received 0.2% CPZ-diet only daily for 30 days, and then fed on a normal ground rodent chow, and administrated the vehicle “used to dissolve SF” intraperitoneally for 14 days.

**Group (3)** CPZ + SF treated rats: rats received 0.2% CPZ-diet only daily for 30 days, followed by intraperitoneal (i.p.) administration of SF, at a dose of 2 mg/ kg, for 14 days.

### Observation of Clinical Signs of CPZ-Induced Toxicity

Home cage observations of the experimental rats were carried out regularly (twice a week until the completion of the experiment); to evaluate clinical signs of CPZ-associated toxicity including behavioral alterations (convulsions, decreased physical activities, lethargy), morphological alterations (e.g., weight loss), and mortality. The bodyweight of rats was measured once a week using a digital balance.

### Preparation of Serum and Tissue Samples

At the end of the experiment, rats were anesthetized using Thiopental sodium (50 mg/kg, i.p.) and blood samples were withdrawn. The serum was separated by centrifugation at 3000 rpm for 20 min (centrifuge 3-18KS, Germany). The rats were then sacrificed under mild anesthesia. The whole liver and heart tissues were dissected; one part was homogenized and aliquots were homogenized in 4 volumes of phosphate-buffered saline (PBS, pH 7.4), and used for biochemical investigations. The other part was weighed, fixed in 10% formal saline, and processed for histological and immunohistochemical analysis of Nrf-2.

### Biochemical Investigations

#### Estimation of Serum Cardiac Troponin I (CTnI)

Serum CTnI was measured using ELISA, according to the manufacturer’s instructions. Results were presented as pg/ml.

#### Estimation of Inflammatory Marker: Interferon-gamma (IFN-γ)

Serum IFN-γ was assayed using ELISA kit, following the manufacturer's protocol. Results were presented as pg/ml protein.

#### Estimation of Pro-inflammatory Marker: Interleukin 1β (IL-1β)

Serum IL-1β was assayed using ELISA kit, according to the manufacturer's guidelines. Results were presented as pg/ml protein.

#### Serum Total Antioxidant Capacity (TAC)

The evaluation of serum TAC is performed by the reaction of antioxidants in the sample with a known amount of exogenous hydrogen peroxide (H_2_O_2_). The antioxidants in the sample eliminate a certain quantity of H_2_O_2_. The residual H_2_O_2_ is estimated by an enzymatic reaction that results in a colored product that could be measured at 505 nm [37].

#### Lipid Peroxidation in the Cardiac and Hepatic Tissues

Evaluating the malondialdehyde (MDA) content, in the cardiac and hepatic tissues, was used to indicate lipid peroxidation. MDA, an end-product of lipid peroxidation, was estimated according to Ohkawa et al. [38]. Briefly, 1000 µl of chromogen was added to 200 µl of tissue homogenate, and the mixture was placed in a boiling water bath for 30 min. In an acidic medium and at a high temperature (95 °C), MDA reacts with thiobarbituric acid and results in a pink-colored product that could be measured spectrophotometrically at 532 nm. The results were expressed as nmol/g tissue.

#### Catalase Activity in the Cardiac and Hepatic Tissues

Catalase reacts with a quantified H_2_O_2_ amount. After one minute, the catalase inhibitor ceased the reaction. Remaining H_2_O_2_ reacts with 3, 5-dichloro-2-hydroxybenzene sulfonic acid (DHBS) and, 4-aminophenazone (AAP), in the presence of peroxidase (HRP), and results in the formation of a colored product with color intensity inversely proportional to catalase activity in the sample [39].

#### Assessment of Liver Functions

Serum alanine amino transaminase (ALT) and serum aspartate amino transaminase (AST) were measured spectrophotometrically by quantitative colorimetric methods according to Reitman and Frankel [40]. Serum alkaline phosphatase (ALP) was measured spectrophotometrically at 510 nm [41].

#### Histopathological Investigation of Heart and Liver Tissues

The liver and heart tissue samples were fixed in a solution of 10% neutral buffered formalin, embedded in paraffin, sectioned at a thickness of 5 μm, and stained with hematoxylin and eosin for regular examination. The sections were analyzed using a light microscope (Leica Microsystems GmbH, Wetzlar, Germany). All standard procedures for sample fixation and staining were conducted according to Culling [42].

#### Immunohistochemical Analysis of Nrf-2

Sections of 5µ thickness were prepared, cardiac or hepatic samples were incubated with primary Anti-Nrf-2 antibody, at 1:500 dilutions, overnight at 4 °C. Tissue sections were washed out by PBS followed by incubation with secondary antibody HRP Envision kit (DAKO) for 20 min; washed out and incubated with diaminobenzidine (DAB) for 15 min. Tissue sections were washed by PBS then counterstained with hematoxylin, dehydrated, cleared in xylene then coverslipped for microscopic examination. Quantification of Nrf-2 was assessed by measuring the % area expression from 5 randomly chosen fields in each section and averaged using image analysis software (Image J, version 1.46a, NIH, Bethesda, MD, USA).

### Statistical Analysis

All values were presented as mean ± standard error of the means (SEM), for *n* = 6 rats of each group. Comparisons between different groups were carried out using one-way analysis of variance (ANOVA) followed by Duncan’s multiple comparison post hoc test. The difference was considered significant when *p*˂0.05. The percent of the change in the value of data for the CPZ + SF group with respect to the negative control or positive control (CPZ) was calculated and presented as a percentage change. ${\text{Percentage of change }}\% \, = \,\left( {{\text{treated}}\;{\text{value}}\; - \;{\text{control}}\;{\text{value}}} \right)/{\text{control}}\;{\text{value}}\; \times \;{1}00$.

## Results

### Effect of CPZ Intoxication and SF Administration in Different Experimental Groups

**Fig. 1:**
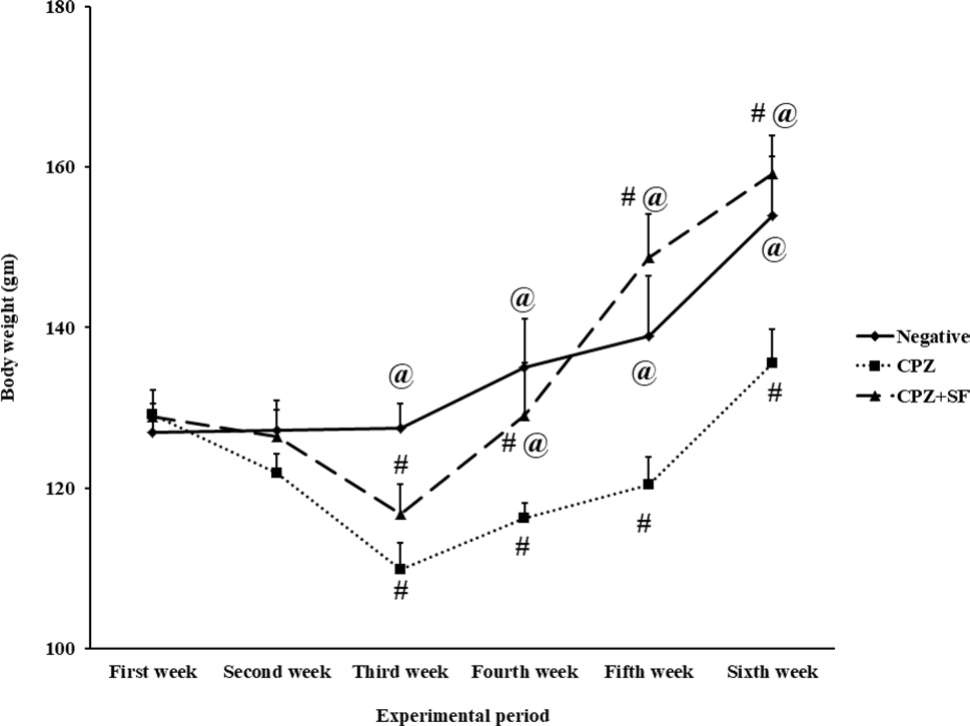
Effect of CPZ administration and SF treatment on body weight of rats in different experimental groups: Negative Control, CPZ (0.2% CPZ-diet for 30 days) group, and CPZ-SF (2 mg/kg/day, for 14 days, i.p.) group. ^#^*p* < 0.05 *vs.* negative control group, ^@^*p* < 0.05 *vs.* CPZ positive control group

Following dietary administration of CPZ, the clinical and behavioral signs, in addition to other toxicity signs including appearance, possible trauma, and mortality were carefully observed. No mortality rate was observed in different groups. Negative control rats and CPZ-induced rats treated with SF demonstrated normal appearance and behavior. On the other hand, CPZ-induced rats demonstrated a clear toxicological profile such as ataxia, lethargy, and decreased physical activities. In addition, CPZ-administrated rats tended to lose weight more quickly. After two weeks of CPZ administration, rats exhibited a significant decrease in body weight, as compared to control rats. However, after CPZ removal from the diet, CPZ-induced rats demonstrated a slight increase in body weight. While both groups of negative control and treated CPZ + SF group continued to gain weight throughout the experimental period, as illustrated in Fig. 1.

### Effect of SF on Cardiotoxicity Marker: CTnI in CPZ-Exposed Rats

**Fig. 2:**
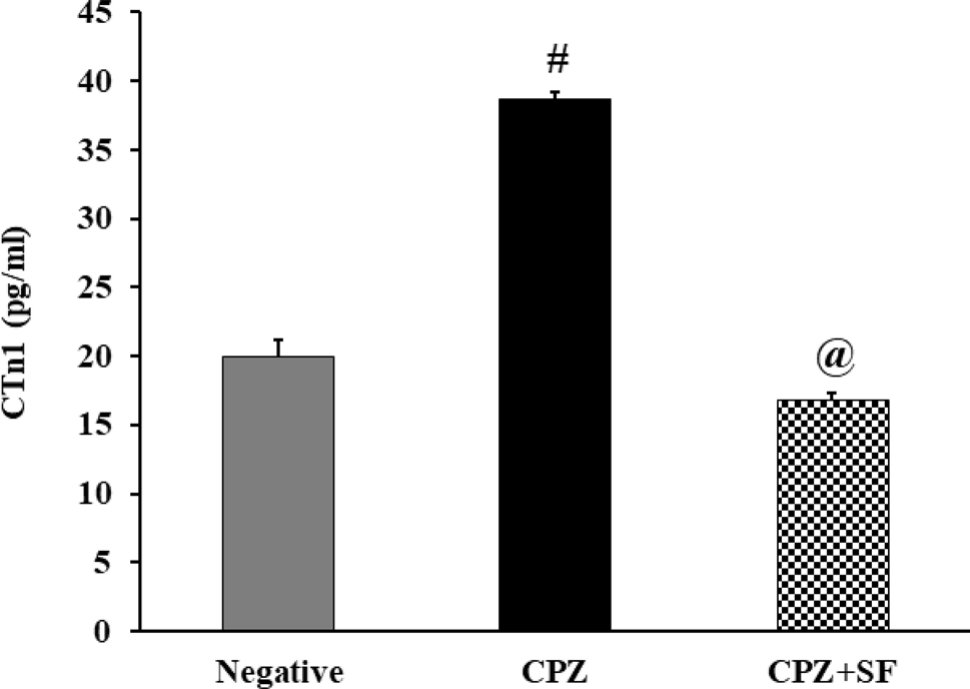
Effect of CPZ administration and SF treatment on serum CTnI in different experimental groups: negative control, CPZ (0.2% CPZ-diet for 30 days) group, and CPZ-SF (2 mg/kg/day, for 14 days, i.p.) group. Each bar represents the mean ± S.E. ^**#**^*p* < 0.05 *vs.* negative control group, ^@^*p* < 0.05 *vs.* CPZ positive control group

CPZ administration enhanced a significant increase in serum CTnI level in CPZ-intoxicated rats by 94.36%, as compared to negative controls. Treatment of CPZ-induced rats with SF caused a significant reduction in CTnI levels by 56.36%, as compared to CPZ rats. These findings indicated the cardiotoxic potential of CPZ and the cardioprotective activities of SF (Fig. 2).

### Effect of SF on Inflammatory Marker: IFN-γ in CPZ-Exposed Rats

**Fig. 3:**
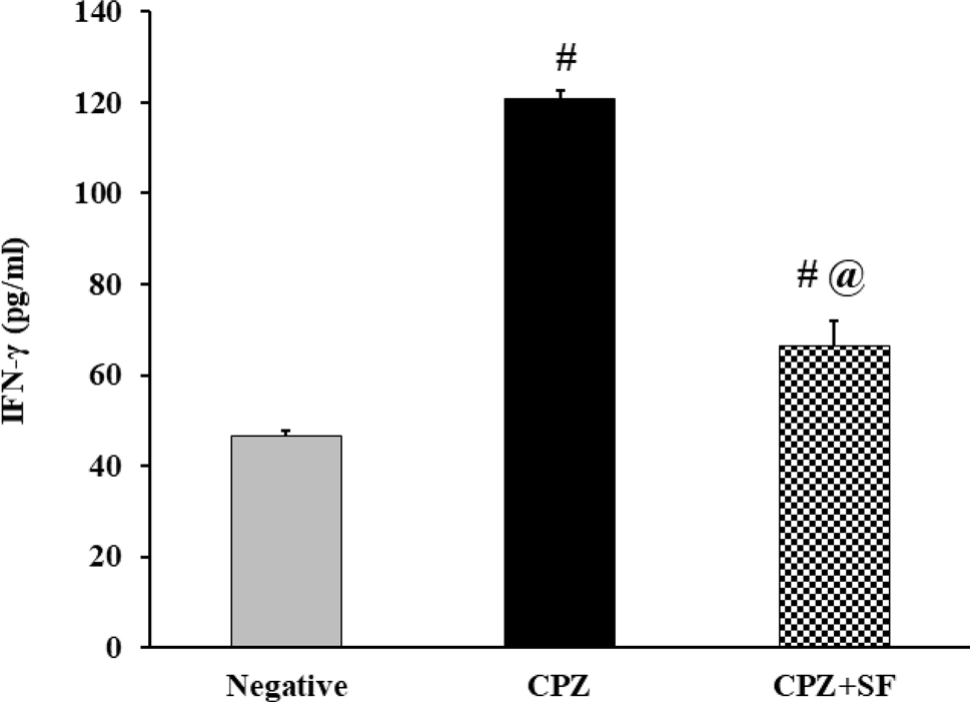
Effect of CPZ and SF treatment on serum IFN-γ in different experimental groups: negative control, CPZ (0.2% CPZ-diet for 30 days) group, and CPZ-SF (2 mg/kg/day, for 14 days, i.p.) group. Each bar represents the mean ± S.E. ^**#**^*p* < 0.05 *vs.* negative control group, ^@^*p* < 0.05 *vs.* CPZ positive control group

In CPZ-exposed rats, CPZ intake caused a significant elevation in serum IFN-γ level by 159.74%, as compared to negative controls. Treatment of CPZ-exposed rats with SF caused a significant decrease in IFN-γ levels by 45%, as compared to CPZ-induced rats. These findings indicated the anti-inflammatory potential of SF (Fig. 3).

### Effect of SF on Pro-inflammatory Marker: IL-1β in CPZ-Exposed Rats

**Fig.4:**
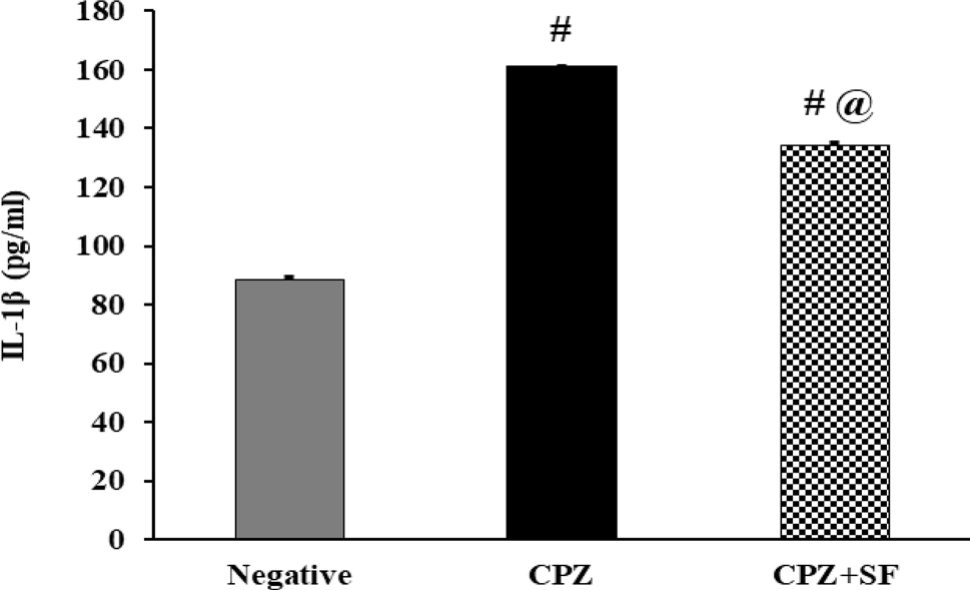
Effect of CPZ and SF treatment on serum IL-1β in different experimental groups: Negative Control, CPZ (0.2% CPZ-diet for 30 days) group, and CPZ-SF (2 mg/kg/day, for 14 days, i.p.) group. Each bar represents the mean ± S.E. ^#^*p* < 0.05 *vs.* negative control group, ^@^*p* < 0.05 vs. CPZ positive control group

In CPZ-intoxicated rats, CPZ exposure resulted in a significant increase in serum IL-1β levels by 82.68%, as compared to negative controls. Treatment of CPZ-induced rats with SF caused a significant reduction in IL-1β levels by 16.59%, as compared to CPZ-fed rats. These findings indicated the anti-inflammatory activity of SF (Fig. 4).

### Effect of SF on Oxidative Stress Status in CPZ-Exposed Rats

**Fig. 5:**
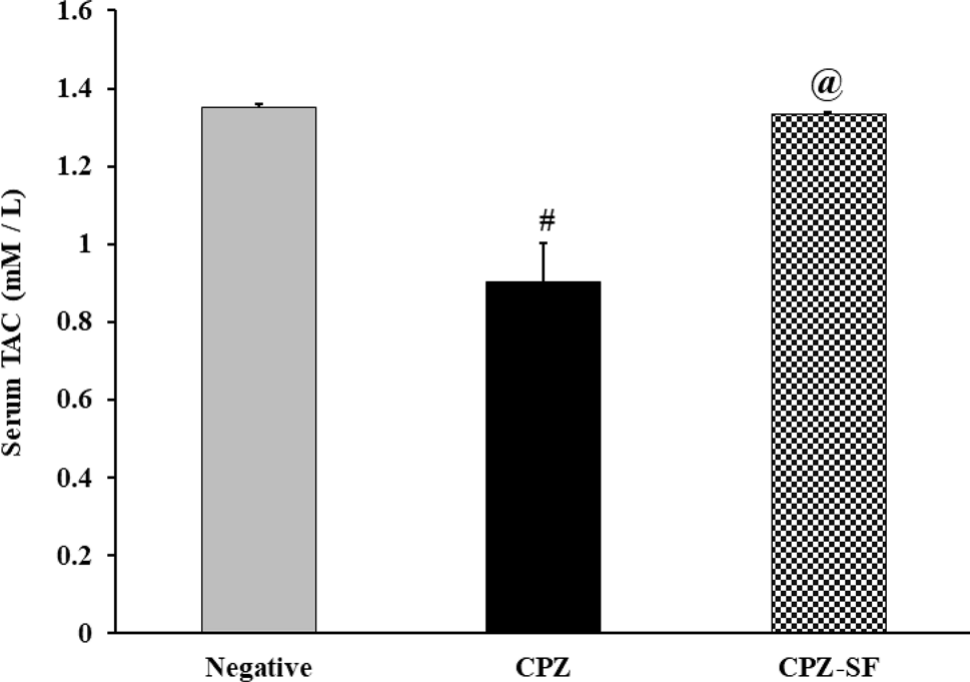
Effect of CPZ administration and SF treatment on serum TAC in different experimental groups: negative control, CPZ (0.2% CPZ-diet for 30 days) group, and CPZ-SF (2 mg/kg/day, for 14 days, i.p.) group. Each bar represents the mean ± S.E. ^#^*p* < 0.05 *vs.* negative control group, ^**@**^*p* < 0.05 vs. CPZ positive control group

**Fig.6:**
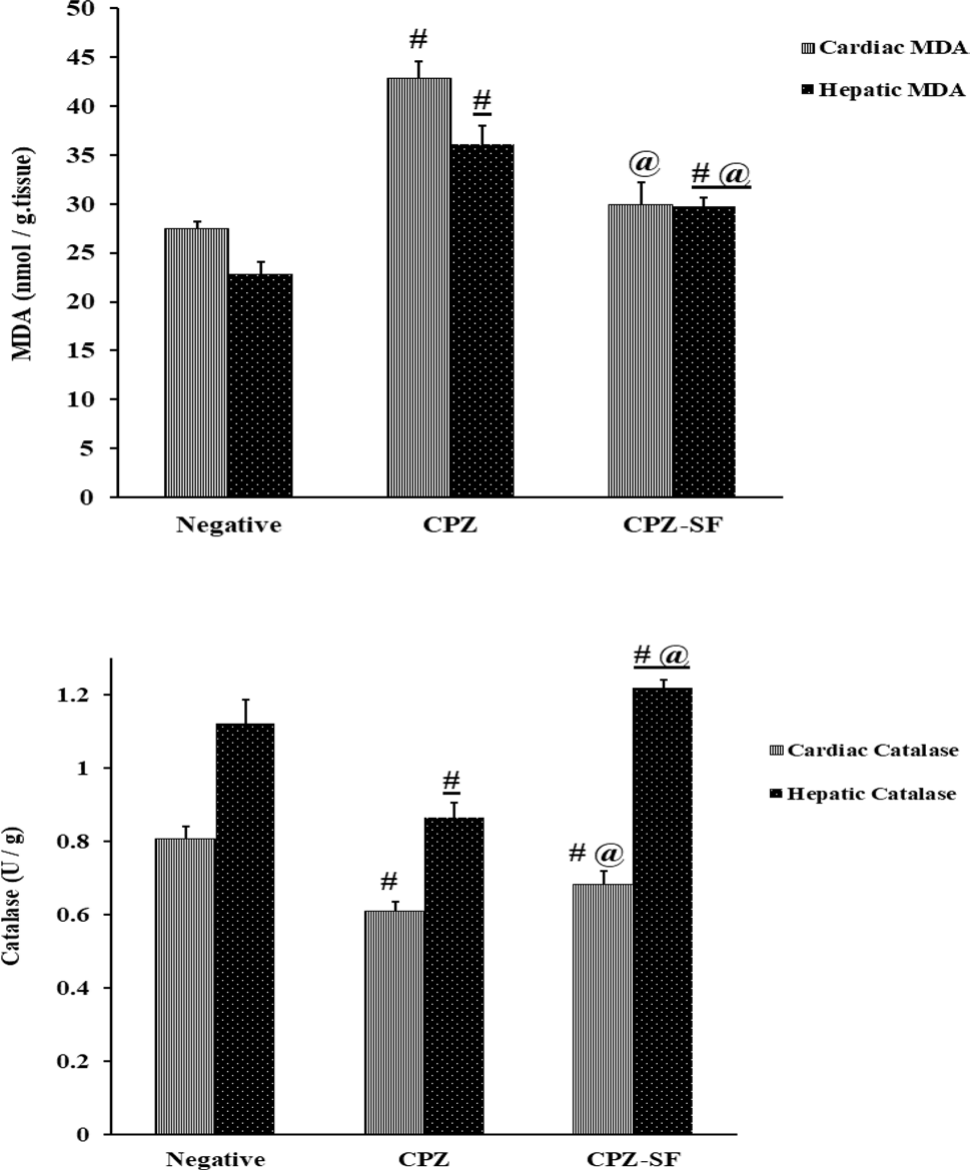
Effect of CPZ administration and SF treatment on cardiac and hepatic MDA contents and catalase activities in different experimental groups: Negative Control, CPZ (0.2% CPZ-diet for 30 days) group, and CPZ-SF (2 mg/kg/day, for 14 days, i.p.) group. Each bar represents the mean ± S.E. ^#^*p* < 0.05 *vs.* negative control group, ^@^*p* < 0.05 vs*.* CPZ positive control group

On the one hand, as compared to negative control rats, CPZ caused a significant reduction in TAC by 33.14%, cardiac catalase by 36.79% and hepatic catalase by 22.8%. Moreover, CPZ administration resulted in a significant elevation in lipid peroxidation in both cardiac and hepatic tissues by 55.87 and 58.73%, respectively. On the other hand, as compared to CPZ-exposed rats, treatment of CPZ-exposed rats with SF resulted in a significant elevation in serum TAC by 47.86%, cardiac catalase by 34%, and hepatic catalase by 40.63%, along with a significant reduction in MDA in both cardiac and hepatic tissues by 30 and 17.7%, respectively. These data demonstrated the anti-oxidative potential of SF (Fig. 5 and 6).

### Effect of SF on Enzymatic Activities of AST, ALT, and ALP

**Fig. 7:**
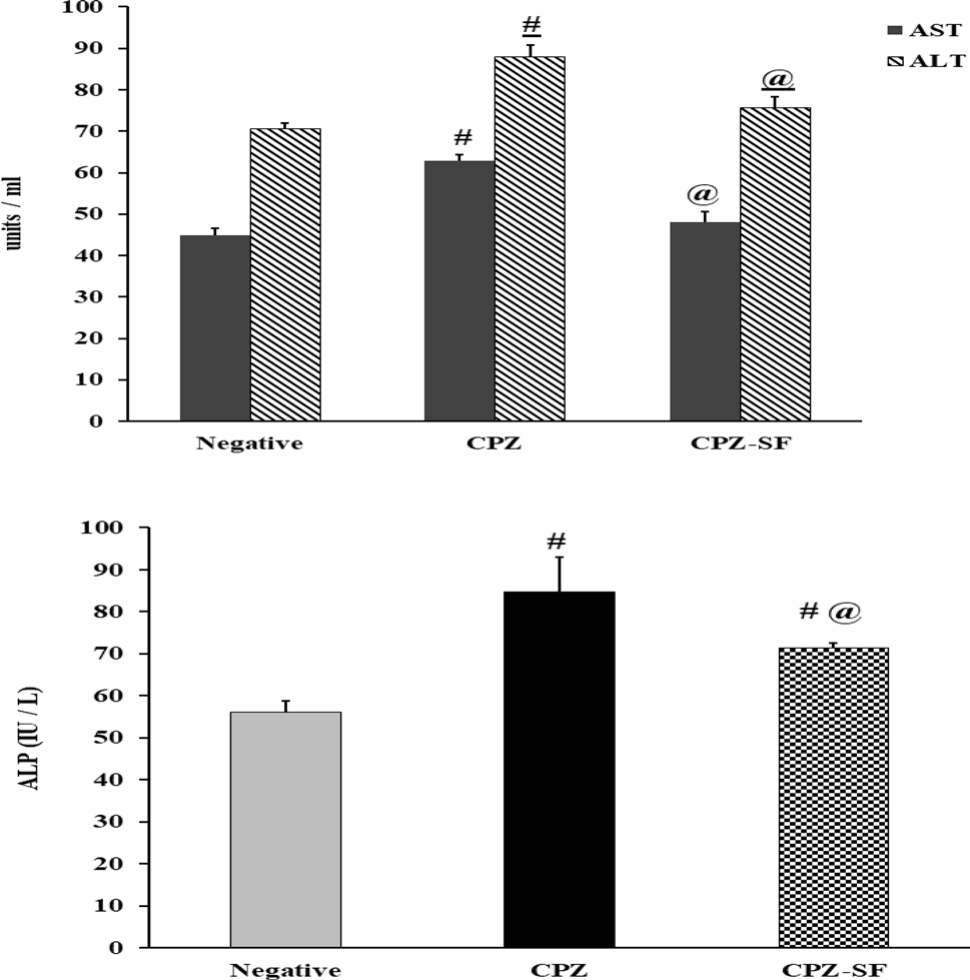
Effect of CPZ administration and SF treatment on AST, ALT, and ALP activities in different experimental groups: negative control, CPZ (0.2% CPZ-diet for 30 days) group, and CPZ-SF (2 mg/kg/day for 14 days, i.p.) group. Each bar represents the mean ± S.E. ^#^*p* < 0.05 *vs.* negative control group, ^@^*p* < 0.05 vs. CPZ positive control group

CPZ-fed rats demonstrated a significant elevation in serum AST, ALT, and ALP by 40.49, 24.63, and 51%, as compared to negative control rats. On the other side, the treatment of CPZ rats with SF caused a significant reduction of 23.83, 14, and 15.7%, as compared to CPZ-induced rats. These data demonstrated the cardioprotective and hepatoprotective activities of SF (Fig. 7).

### Effect of SF on CPZ-Induced Histological Architecture of the Heart and Liver

**Fig. 8:**
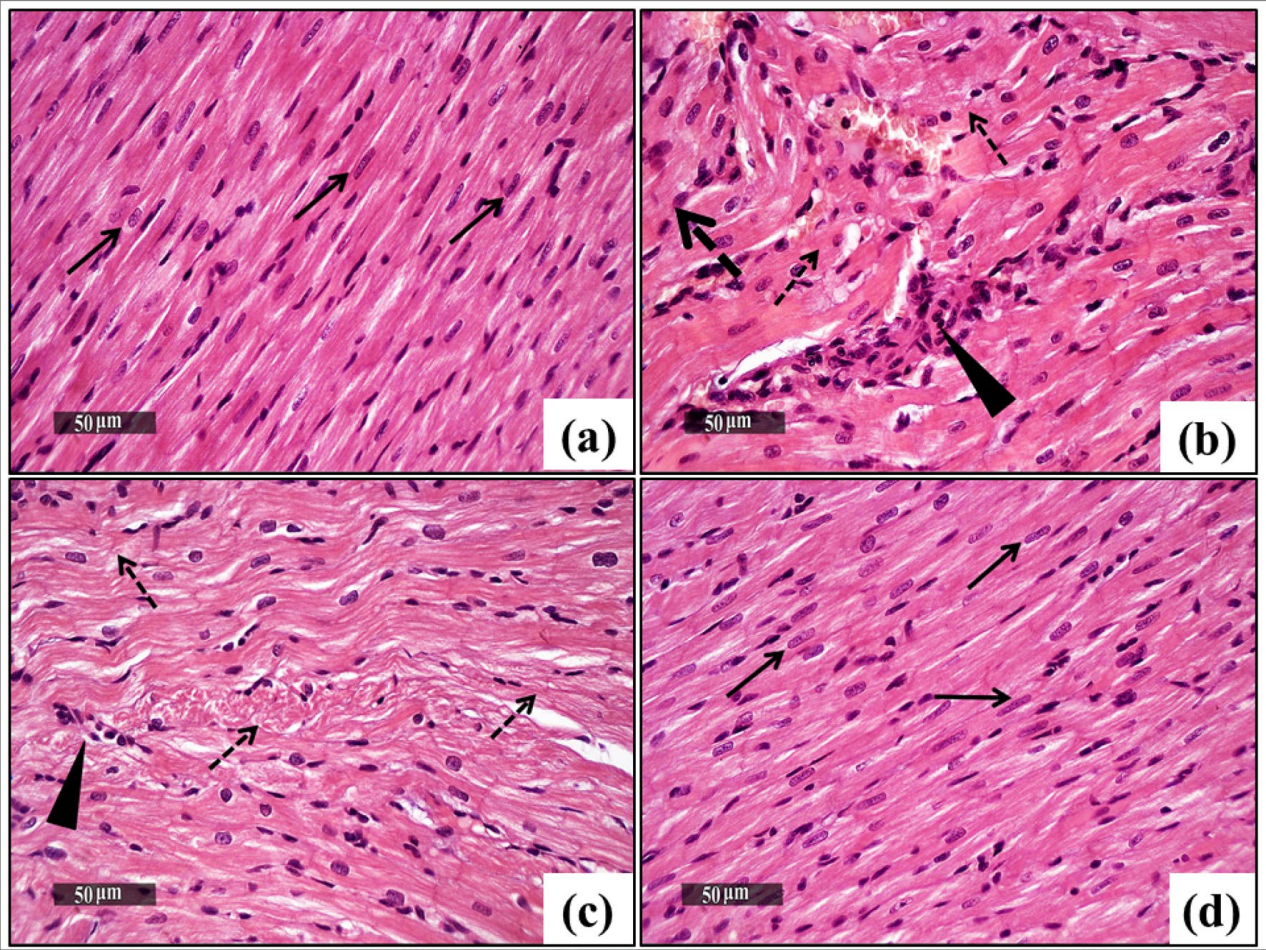
Effect of CPZ administration and SF treatment on cardiac histoarchitecture in different experimental groups: negative control, CPZ (0.2% CPZ-diet for 30 days) group, and CPZ-SF (2 mg/kg/day, for 14 days, i.p.) group, (scale bar: 50 μm). Cardiac tissues of negative control rats (**a**) demonstrated normal cardiomyocytes with intact subcellular details (arrow). CPZ-intoxicated cardiac tissues demonstrated degenerated cardiomyocytes (dashed arrow) and infiltration of inflammatory cells (arrowhead) (**b**, **c**). CPZ + SF treated group showed almost normal histological features with well-defined cardiomyocytes (**d**). Preference for color: online

**Fig. 9:**
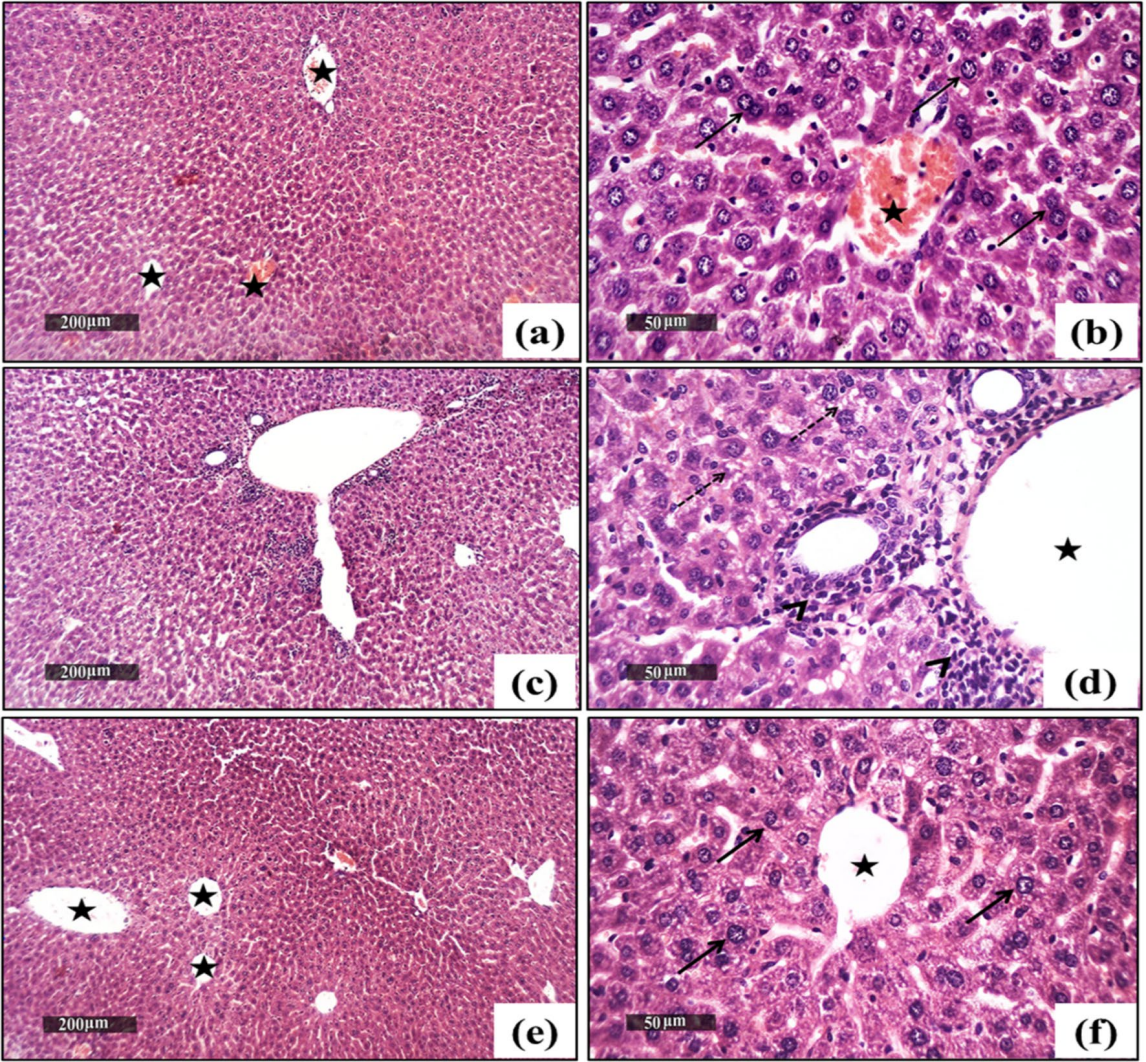
Effect of CPZ administration and SF treatment on hepatic histoarchitecture in different experimental groups: negative control, CPZ (0.2% CPZ-diet for 30 days) group, and CPZ-SF (2 mg/kg/day, for 14 days, i.p.) group, (scale bar: 200 and 50 μm). Normal control hepatic tissues exhibited normal hepatocytes with intact parenchyma (arrow) and intact hepatic vasculatures (star) (**a**, **b**). CPZ-intoxicated hepatocytes demonstrated showed moderate dilatation of hepatic vasculatures (star) and moderate periportal inflammatory cells infiltrates (black arrow), and slight hepatic degeneration (dashed arrow) (**c**, **d**). CPZ + SF treated hepatic tissues showed moderate dilatation of hepatic vasculatures were shown (star) with mild hepatocellular vacuolar degenerative alterations (dashed arrow) (**e**, **f**). Preference for color: online

Histopathological investigations of cardiac and hepatic tissues of different experimental groups are illustrated in Figs. 8 and 9.

Concerning the cardiac histoarchitecture, normal control samples demonstrated normal histological features of the cardiac wall with many apparent well-organized, striated and branched cardiomyocytes with intact subcellular details (arrow) and intact vasculatures (Fig. 8a). By contrast, CPZ-induced hearts exhibited focal areas of fragmented and degenerated cardiomyocytes (dashed arrow) with the mild widening of intercellular spaces accompanied by mild occasional interstitial inflammatory cells infiltrates (arrowhead) (Fig. 8b, c). Meanwhile, CPZ + SF treated hearts showed almost intact histological features without abnormal histological changes records (Fig. 8d).

Concerning the hepatic histoarchitecture, Normal control hepatic samples demonstrated normal histological features of liver parenchyma with many apparent intact well-organized hepatocytes with intact subcellular details (arrow), intact hepatic vasculatures (star), as well as hepatic sinusoids were shown without abnormal changes records (Fig. 9a, b). In contrast, CPZ-intoxicated hepatic tissues showed significant dilatation of hepatic vasculatures (star) and sinusoids accompanied by periportal inflammatory cell infiltrates (arrow), and few records of hepatocellular degenerative changes (dashed arrow) (Fig. 9c, d). On the other side, CPZ + SF treated livers showed intact subcellular details (arrow), and intact hepatic vasculatures (star) with minimal records of inflammatory cell infiltrates (Figs. 9e, f).

### Effect of SF on Immunohistochemical Expression of Nrf-2 in Cardiac and Hepatic Tissues

**Fig. 10:**
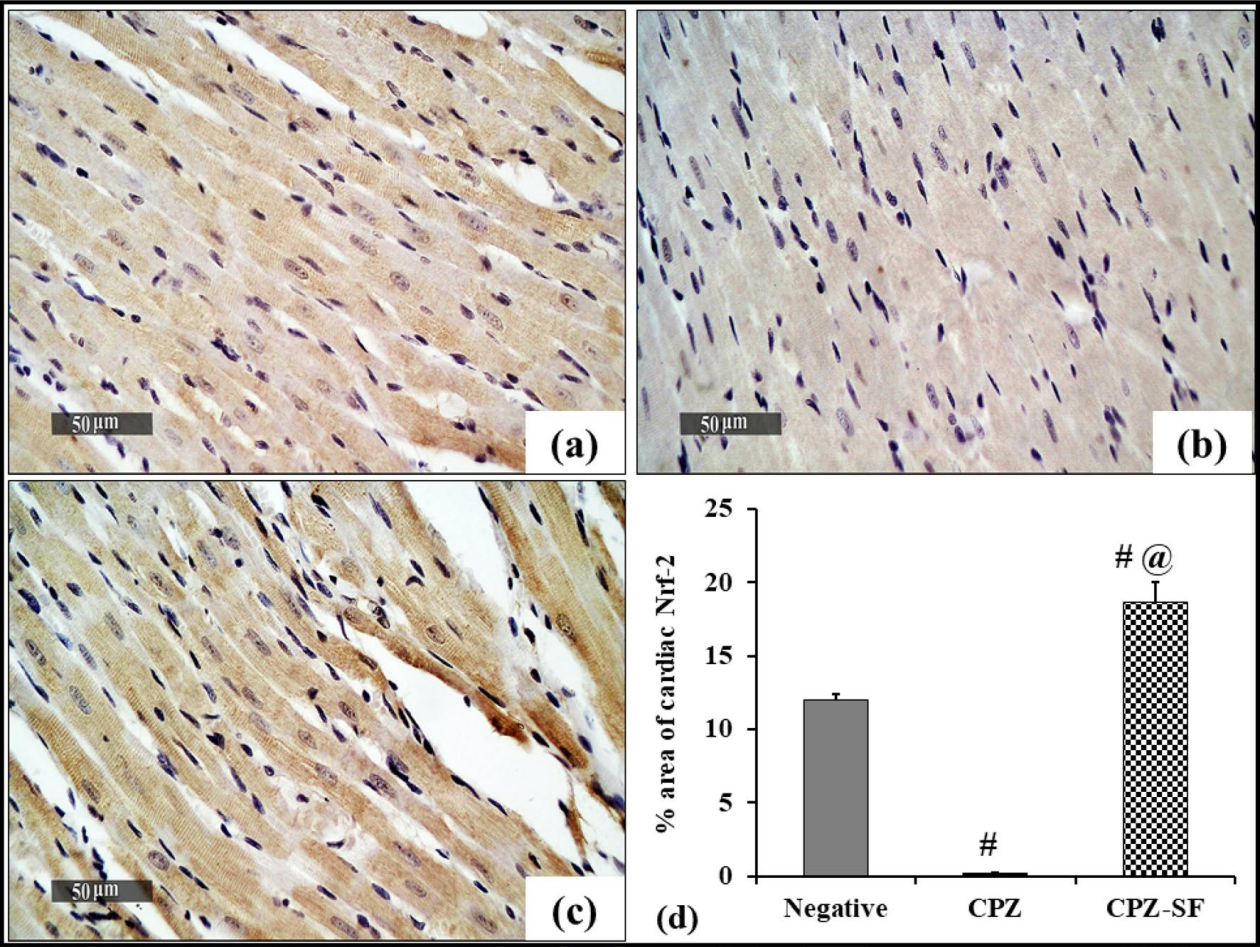
Effect of CPZ administration and SF treatment on immunostaining of Nrf-2 in the cardiac tissues of different experimental groups: negative control, CPZ (0.2% CPZ-diet for 30 days) group, and CPZ-SF (2 mg/kg/day, for 14 days, i.p.) group, (scale bar: 50 μm). Heart tissue of the negative control group demonstrated moderate immunoexpression of Nrf-2 with a significant increase of positive immunostaining cells (**a**). CPZ-intoxicated cardiac tissues showed no immunoexpression of Nrf-2 (**b**). CPZ + SF treated cardiac tissues exhibited strong immunoexpression of Nrf-2 with a significant increase of positive immunostaining cells (**c**). The immune staining area (%) of Nrf-2 in cardiac tissues is illustrated in **d**. Each bar represents the mean ± S.E. ^**#**^*p* < 0.05 *vs.* negative control group, ^@^*p* < 0.05 vs. CPZ positive control group. Preference for color: online

**Fig. 11:**
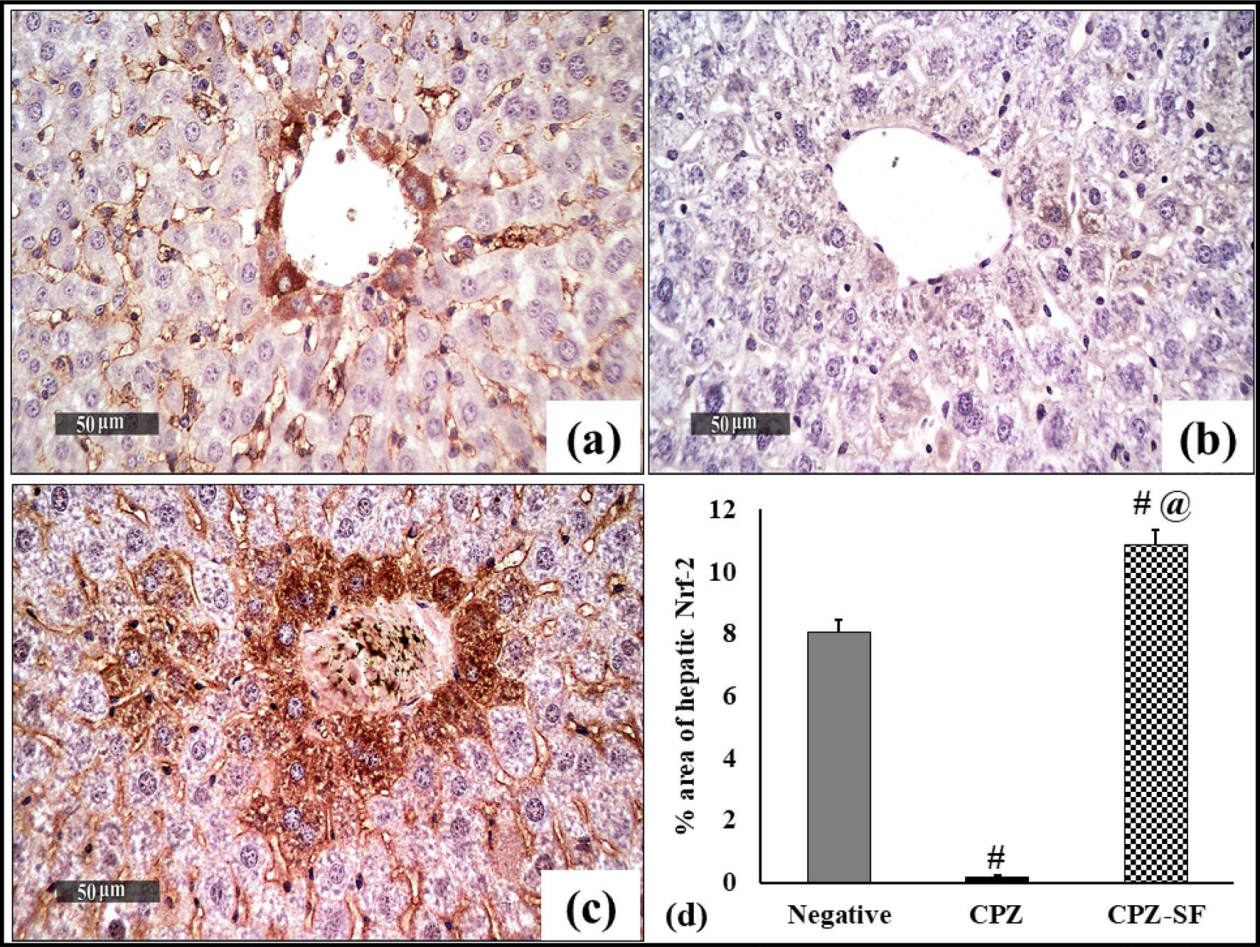
Effect of CPZ administration and SF treatment on immunostaining of Nrf-2 in the hepatic tissues of different experimental groups: negative control, CPZ (0.2% CPZ-diet for 30 days) group, and CPZ-SF (2 mg/kg/day, for 14 days, i.p.) group, (scale bar: 50 μm). Hepatic tissue of the negative control group demonstrated moderate immunoexpression of Nrf-2 (**a**). CPZ-intoxicated liver tissues showed weak immunoexpression of Nrf-2 (**b**). CPZ + SF treated hepatic tissues exhibited strong immunoexpression of Nrf-2 with a significant increase of positive immunostaining cells (**c**). The immune staining area (%) of Nrf-2 in liver tissues is illustrated in **d**. Each bar represents the mean ± S.E. ^**#**^*p* < 0.05 *vs.* negative control group, ^@^*p* < 0.05 vs. CPZ positive control group. Preference for color: online

Immunohistochemical expressions of Nrf-2 in the cardiac and hepatic tissues are depicted in Fig. 10 and 11. Briefly, the normal control rats revealed a moderate immunoexpression of Nrf-2 in both heart and liver tissues (Figs. 10 and 11a), respectively. In contrast, CPZ-induced cardiac and hepatic tissues exhibited no immunoexpression of Nrf-2; indicating the inhibitory influence of CPZ on the immunoexpression of Nrf-2 (Figs. 10, 11b). On the other side, CPZ + SF treated cardiac and hepatic tissues demonstrated strong positive immunostaining cells of Nrf-2 (Figs. 10, 11c); signifying that SF upregulated the immunoexpression of Nrf-2. Figures 10 and 11d described the immunostaining area (%) of Nrf-2 immunoexpression in the heart and liver of rats from different experimental groups.

## Discussion

CPZ is a well-known neurotoxicant that is commonly used to induce demyelination and oligodendroglial death by stimulating mitochondrial dysfunction and provoking oxidative stress by increasing mitochondrial generation of reactive oxygen species (ROS) [1, 43]. Herein, we investigated CPZ as a cardiotoxic and hepatotoxic chemical and examined the potential protective activities of SF against CPZ-induced cytotoxicity and hepatotoxicity via suppressing oxidative stress and modulating inflammation.

The animal weight is an essential “variable” for reliable CPZ-provoked toxicity [44]. The first effect observed by the CPZ administration was body weight loss after two weeks; the body weight of CPZ-fed rats exhibited a significant reduction of about 14% in the 3^rd^ and 4^th^ weeks of CPZ intake. Our results run in agreement with Akyuz and Villa [8], the loss of about 15% is a hallmark of the CPZ-induced demyelination. This long-term intoxication (four weeks) caused the morphological signs of CPZ-associated neurotoxicity in most cases accompanied by weight loss and characteristic movement disorders of the CPZ-induced animals [45, 46]. This reduction in the body weight of CPZ rats indicates reduced food intake and disruption of normal energy flow, as compared to negative controls; this might be due to the unpleasant odor or taste of CPZ and its impact on the hepatic function [47, 48]. In addition, copper chelation could be another cause of CPZ-induced weight loss, considering Cu-deficiency in rats [49]. Cessation of CPZ administration resulted in the restoration of body weight gain but at a slower rate, our results run in agreement with Babbs et al. [50] that showed recovery of weight loss following replacement of the CPZ-diet with the regular diet. On the other hand, treatment of CPZ rats with SF was able to restore body weight gain, signifying restoration of appetite upon cessation of CPZ administration. SF exhibited the potential of restoring body weight gain, ameliorating the neurotoxicity signs, and improving the clinical symptoms of CPZ intoxication. These finding showed the lowering body weight influence of CPZ, as well as, the therapeutic (gaining body weight) potential of SF. Our results highlighting the importance of body weight as a parameter of clinical appearance of toxicity.

Dietary administration of CPZ to rats is capable of initiating a cascade of oxidoinflammatory events; CPZ-provoked oxidative stress manifested as declined serum TAC levels, decreased cardiac and hepatic catalase activities, along with a significant increment in lipid peroxidation in cardiac and hepatic tissues. This oxidative potential of CPZ could be ascribed to its nature as a Cu-chelator that interferes with the activity of Cu-dependent enzymes “cuproenzymes” such as “cytochrome oxidase” that is particularly implicated in mitochondrial respiration [43]; mitochondria are the major organelles responsible for ROS propagation, which are readily scavenged by the antioxidative enzyme “catalase”. Therefore, CPZ-induced declined catalase activity promoted the oxidative impact.

CPZ-induced oxidative stress in both the hepatic and cardiac tissue, especially lipid peroxidation, might be involved in the disruption of the cellular membranes and the release of transaminases [51]; therefore, restoring these enzymatic activities to their normal levels is associated with the attenuation of the hepatotoxicity and restoration of the cardiac function. The release of intracellular ALT and AST could enhance the status of inflammation and hepatic fibrosis [52]. AST could be used as a marker of both cardiotoxicity and hepatotoxicity; CPZ administration caused significant increments in AST (40.49%), ALT (24.63%), ALP (51%), and CTn1 (94.36%), as compared to negative control rats. CPZ caused a significant increase in AST, a serum indicator of “myocardial injury” [53]; disruption of cardiomyocytes caused the leakage of AST, which normally exists in the cytoplasm of cardiomyocytes [54]. Furthermore, high serum levels of CTn1 and AST and induced ROS generation are considered key hallmarks of cardiotoxicity [51]. Elevated CTnI levels indicate irreversible cardiac damage and necrosis of cardiomyocytes [55]. This highlights the potential use of CTn1 as a highly specific and sensitive cardiac diagnostic biomarker for myocardial necrosis [56].

Furthermore, CPZ enhanced a significant increase in serum levels of the inflammatory marker IFN-γ (159.74%) and the pro-inflammatory marker IL-1β (82.68%), as compared to negative control rats. This increased level of the inflammatory IFN-γ might be attributed to the release of IFN-γ from the disrupted hepatocytes; IFN-γ further provoked the activation and proliferation of hepatic stellate cells (HSCs) [57]. IFN-γ is a crucial regulator of the complex process of inflammatory mediators in cardiac pathological conditions that includes myocardial infarction or myocarditis [58]. In addition, CPZ intake upregulated the expression of several genes of inflammation [59]; Il-1β is upstream of many inflammatory mechanisms [60]. Our results run in agreement with Hillis et al. [61] and Mason et al. [59] that demonstrated enhanced IL-1β expression in the CPZ-induced animals; Mason et al. [59] found that expression of IL-1β was gradually increased in the 1^st^ week of CPZ intake, then robustly upregulated in the 3^rd^ week till the 6^th^ week. These biochemical results were supported by histopathological analysis of the heart and liver tissues; which demonstrated that CPZ-provoked structural alterations and induced inflammation in both tissues. Furthermore, CPZ-induced oxidative stress in both tissues was evidenced by the declined immunoexpression of Nrf-2. The disruption of Nrf-2 signaling stimulates susceptibility to oxidative and electrophilic stresses, as well as, to inflammation [62].

On the other side, treatment of CPZ-exposed rats with SF caused a significant reduction in AST, ALT, and ALP; in addition, CPZ-induced cardiac dysfunction could be reversed by SF treatment as demonstrated by decreased serum CTn1 levels. These findings run in agreement with previous studies [25, 55, 63]. This cardioprotective action of SF might be ascribed to its anti-oxidative potential and anti-lipid peroxidation activity that resulted in “membrane protection” and suppressed the cellular leakage of transaminases and CTn1, this run in agreement with several studies [28, 29, 35]. These hepatoprotective and cardioprotective actions were accredited to the up-regulation of hepatic and cardiac Nrf-2 and heme oxygenase-1 (HO-1) expression by SF [64], as evidenced by elevated Nrf-2 immunoexpression in both cardiac and hepatic tissues; suggesting that Nrf-2 is a therapeutic target in inflammation-associated conditions. Nrf-2 activated cytoprotective genetic function might act synergistically to regulate innate immune response and to inhibit the up-regulation of pro-inflammatory gene expression [65].

Nrf-2 activation restores the balance between oxidants and antioxidants and plays a key role in the expression of several antioxidant genes including catalase [62, 66, 67]. Treatment of CPZ-intoxicated rats with SF counteracted the enhanced oxidative stress in both cardiac and hepatic tissues. SF exhibited the potential to enhance the expression of detoxification or defensive enzymes via the Keap1/Nrf2/ARE signaling pathway [30]. Immunohistochemical results demonstrated Nrf-2 activation upon treatment with SF; this could be ascribed to the potential of Nrf-2 to regulate the up-regulation of detoxification genes to activate a cytoprotective response, through mediating the inactivation of oxidants and favoring the synthesis of antioxidants [68]. Moreover, the translocation of Nrf-2 to the nucleus enables its interaction with the antioxidant response element (ARE) to boost the cytoprotective “anti-oxidative” gene expression, such as HO-1 and NAD(P)H: quinone oxidoreductase 1 (NQO1), to produce catalase to exert anti-oxidative function [69]. Herein, we estimated the catalase activities and evaluated the immunostaining % of Nrf-2; as Nrf-2 controls the expression of antioxidant catalase, which is significantly influenced by redox status, as evidenced previously [70], although other antioxidant enzymes might be involved.

Nrf-2 is vital for the maintenance of redox signaling in response to stress [71]. Under stress conditions such as CPZ-induced toxicity, Nrf-2 loses its DNA binding to ARE, however, this loss of Nrf-2/ARE binding could be reversed by using “Nrf-2 agonists” such as SF [72, 73]; suggesting that Nrf-2-mediated survival pathways are reversible and responsive by using effective agonists of Nrf-2 such as SF, which act as “indirect antioxidant” with anti-inflammatory potential [30, 74]. The inhibitory potential of SF on abnormally activated inflammation is related to the activation of Nrf-2-mediated signaling pathways [75]. It was evidenced that Nrf-2 is one of the most vital defense mechanisms against oxidative and/or electrophilic stresses [71, 76]; activation of Nrf-2 signaling has conferred protection against myocardial ischemia/reperfusion (I/R) injury [77] and in isolated rat hearts [78]. It was found that SF-based Nrf-2 activation protects the myocardium from Angiotensin II-induced cardiomyopathy [79]. In contrast, the absence of Nrf-2 inhibited the expression of antioxidant genes in the myocardium, causing fibrosis and apoptosis [80, 81]; downregulation of Nrf-2 is correlated with redox-sensitive vascular dysfunction [82]. Moreover, SF is capable of interacting with cysteine residues on Kelch-like ECH-associated protein 1 (Keap1) “cysteine-rich adaptor protein”, enhancing its dissociation from Nrf-2 and enhancing the nuclear accumulation of Nrf-2 that subsequently enhances the genetic expression of phase II detoxification enzymes [83]. Therefore, the “Nrf-2 activators” could exhibit cardio-therapeutic applications where inflammation and oxidative injury play key roles [84].

The anti-oxidative and anti-inflammatory activities of SF are strongly linked to the cardioprotective and hepatoprotective activities. Experimentally, dietary SF protects against a wide variety of hepatotoxic chemicals [63, 64, 85]. SF was able to reduce ALT, AST, and ALP, decrease lipid peroxidation, and enhance TAC levels and catalase activities. Furthermore, SF administration reduced CPZ-induced oxidative stress in both cardiac and hepatic tissues. Our results run in accordance with Greco et al. [86] and Xu et al. [70] that showed that treatment of induced rats with SF increases Nrf-2-mediated antioxidant defenses; this might be explained by the ability of SF as Nrf2 regulator to control CPZ intoxication, possibly by potentiating catalase function, signifying that the re-activation of the “Nrf2-catalase signaling pathway” and enhancing the “peroxidase activity” of catalase. SF demonstrated anti-inflammatory and anti-oxidative activities as demonstrated previously [87–89]. SF was capable of reducing both IL-1β and IFN-γ levels, this run in agreement with several studies [90–93], signifying the role of SF as an antioxidant bioactive molecule with anti-inflammatory potential.

This study did not include negative control rats that were administrated SF alone as several studies showed the non-toxic potential of SF at a dose range of 0.5 to 12.5 mg/kg/day either orally [63, 88] or intraperitoneally [94, 95]. This step was taken as an ethical measure to decrease the number of sacrificed rats. The present study illustrated the protective role of SF against CPZ-induced cardiotoxicity and hepatotoxicity primarily via Nrf-2 activation and the accompanied anti-inflammatory and anti-oxidative functions. This study is the first to investigate the protective potential of SF against CPZ-induced cardiotoxicity and hepatotoxicity. Further studies are required to investigate the molecular mechanisms and the mechanistic pathways underlying the protective potential of SF as well as the cytotoxic potential of CPZ. However, the efficient clinical use of Nrf-2 activators to manage inflammatory diseases requires further validation to avoid the side effects of Nrf-2 activation.

## Data Availability

The datasets generated during and/or analyzed during the current study are included within the manuscript.

## Abbreviations

ALP: Alkaline phosphatase
ALT: Alanine amino transaminase
ARE: Antioxidant response element
AST: Aspartate amino transaminase
CPZ: Cuprizone
CTn1: Cardiac troponin 1
Cu: Copper
IFN-γ: Inflammatory interferon-gamma
IL-1β: Interleukin 1β
MDA: Malondialdehyde
Nrf-2: Nuclear factor erythroid 2-related factor 2
ROS: Reactive oxygen species
SF: Sulforaphane
TAC: Total antioxidant capacity
